# Hybrid mechanical metamaterials: Advances of multi-functional mechanical metamaterials with simultaneous static and dynamic properties

**DOI:** 10.1016/j.heliyon.2025.e41985

**Published:** 2025-01-17

**Authors:** Ana Carolina Azevedo Vasconcelos, Dingena Schott, Jovana Jovanova

**Affiliations:** Mechanical Engineering Faculty, Delft University of Technology, Delft, the Netherlands

**Keywords:** Vibration attenuation, Energy absorption, Multi-functionality, Hybrid mechanical metamaterials, Engineering problems

## Abstract

Mechanical metamaterials are architected structures with unique functionalities, such as negative Poisson's ratio and negative stiffness, which are widely employed for absorbing energy of quasi-static and impact loads, giving improved mechanical response. Acoustic/elastic metamaterials, their dynamic counterparts, rely on frequency-dependent properties of their microstructure elements, including mass density and bulk modulus, to control the propagation of waves. Although such metamaterials introduced significant contribution for solving independently static and dynamic problems, they were facing certain resistance to their use in real-world engineering problems, mainly because of a lack of integrated systems possessing both mechanical and vibration attenuation performance. Advances in manufacturing processes and material and computational science now enable the creation of hybrid mechanical metamaterials, offering multifunctionality in terms of simultaneous static and dynamic properties, giving them the ability of controlling waves while withstanding the applied loading conditions. Exploring towards this direction, this review paper introduces the hybrid mechanical metamaterials in terms of their design process and multifunctional properties. We emphasize the still remaining challenges and how they can be potentially implemented as engineering solutions.

## 1 Introduction

Dynamic loads significantly contribute to the behavior and performance of engineered structures, often leading to undesirable structural responses characterized by nonlinearities, resonances, and structural failure. Understanding and mitigating these responses are crucial for ensuring the safety and reliability of engineered structures across diverse fields, including civil infrastructure, aerospace, mechanical systems and offshore installations.

Shielding structures relying on large plastic deformation or significant cracking are conventionally utilized to protect other engineered structures from diverse loading conditions. Cellular structures, for instance, have been widely used in aerospace and automotive industries due to their improved strength-to-relative density ratio and high energy absorption capability in comparison to their constituent material [1], [2], although suffering from crushing [3]. Relying on irreversible mechanisms may hinder material reuse under repeated loads, significantly escalating the costs of manufacturing new shielding materials and generating material waste. This has instigated the search for shielding structures that are capable of suppressing the transmission of mechanical waves caused by such loads, while retaining their structural integrity.

Decades ago, researchers unveiled metamaterials (MM)s, which are materials exhibiting unique properties capable of manipulating electromagnetic and optical waves [4], [5]. This breakthrough boosted the development of a novel subset of MMs for absorbing mechanical energy, which has been named as mechanical metamaterials (MMM)s. MMMs show improved mechanical properties compared to nature materials, such as negative Poisson's ratio and negative stiffness, stemming from the strategic design of their building blocks or unit cells. Such unique features have enabled the development of light-weight MMM structures with high-strength capacity [6], which have been widely applied in aircraft structures.

Another application of MMMs includes the control of mechanical vibrations, such as airborne vibrations and structural vibrations. These MMMs are usually referred to as acoustic/elastic metamaterials (AEMM)s and their controlling capacity stems from two mechanisms: Bragg scattering and local resonance [7]. AEMMs exhibiting the Bragg scattering mechanism are usually called phononic crystals (PnC)s, which consist of periodically arranged microstructures–or unit cells–serving as scatterers. Propagating acoustic/elastic waves interact with these scatterers and produce dispersion relations showing band gaps–or attenuation zones–in specific frequency ranges. In these band gaps, waves with wavelength in the order of unit cell size are attenuated, while outside the range, waves propagate without loss. Locally-resonant AEMMs exhibit frequency-dependent mechanical properties due to the local resonators arranged in their micro-structure. This enables the mechanical wave control in specific frequency ranges called “resonant band gap”. The induced resonant band gaps originate from the interference between incoming waves and the waves re-radiated from the local resonators, also named as Fano-type interference [8]. The resonance can be adjusted by modifying the resonator's mass or stiffness. Unlike Bragg scattering, this approach enables the formation of subwavelength band gaps, facilitating low-frequency wave mitigation for diverse applications, such as vibration control [9], [10], blast loading resistance [11], sound isolation [12], and seismic isolation [13].

**Figure 1:**
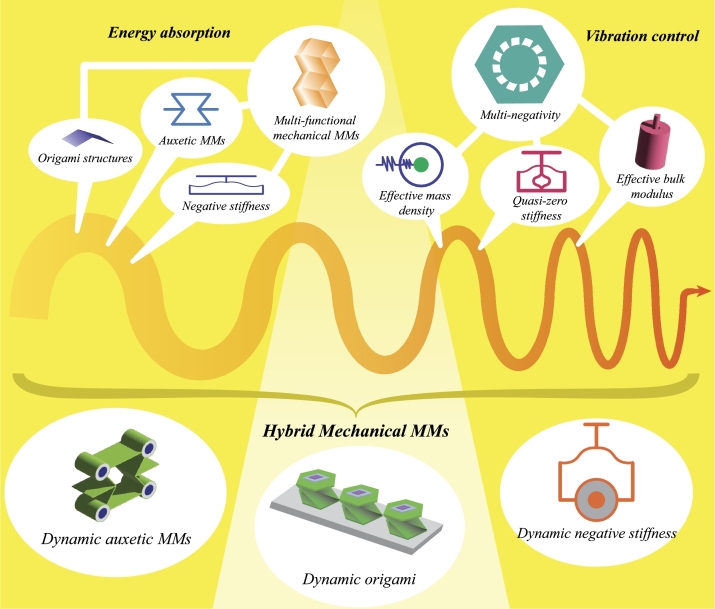
Hybrid mechanical metamaterials origin: the combination of energy-absorbing mechanisms from mechanical metamaterials and vibration-controlling mechanisms of elastic/acoustic metamaterials.

Although MMMs have shown important contributions to energy absorption in static problems and vibration control in dynamic problems, complex applications involving static and dynamic load conditions demand MMMs integrating multiple functionalities. For example, MMMs subjected to dynamic loads may require vibration attenuation performance to prevent resonance in protected structure, while concurrently exhibiting energy absorption capabilities for load-bearing purposes. The recent developments in computational design tools and additive manufacturing (AM) processes have facilitated the investigation and fabrication of complex designs with enhanced functionalities [14]. Among these designs, hybrid mechanical metamaterials (HMMM)s are emerging as a promising solution for such complex problems, as they integrate multiple mechanisms to achieve diverse functionalities (see Fig. 1).

One of the biggest challenges of the current MMM designs are their implementation in real-world engineering problems faced in automotive and aerospace, infrastructure design, underwater acoustics, among others. This trend, however, may change with the development of HMMMs, once they are moving towards single-integrated systems that target different properties. Thus, comprehending the individual mechanisms governing energy absorption and vibration control is essential for tailoring HMMMs for specific applications. While numerous review papers have documented the mechanisms of MMMs [15] and AEMMs [16], an overview of the existent HMMMs and emerging design trends for engineering applications remains absent.

This review paper elucidates traditional MMM mechanisms for energy absorption and vibration isolation, how these mechanisms are integrated into HMMM designs to overcome the challenges faced by traditional MMMs, and explores future directions for their implementation as engineering solutions. Section 2 introduces MMM mechanisms for energy absorption capability, while Section 3 presents AEMMs containing unusual effective values of mass and stiffness. Section 4 highlights the design process of HMMMs and their potential multifunctional properties. The section ends with a discussion of the next generation of HMMMs and how they can be implemented in engineering applications.

## 2 Mechanical metamaterials for energy absorption

The mechanical properties of energy-absorbing materials, such as cellular materials (for instance, foams), polymers and gels, are crucial for engineering applications where structures face various loading conditions. However, cellular materials have practical limitations as they often rely on non-recoverable absorption mechanisms, leading to plastic deformation. This feature may prove unsuitable for applications involving repetitive loads and can result in increased material wastage.

In response to the demand for materials that can endure challenging loading conditions, researchers have developed mechanical metamaterials characterized by exceptional energy absorption and high specific strength, which stem from the microstructure geometry–also referred as unit cell. The energy-absorbing performance is attributed to the deformation modes and loading conditions–notice that material properties also play an important role, however, only the geometric aspects will be discussed here. In this context, we dedicate this section to summarize and discuss the energy absorption mechanisms of mechanical metamaterials. Then, the response of such mechanisms under diverse load conditions is investigated. Finally, the unique properties of mechanical metamaterials are presented according to the designed mechanisms and loading conditions.

### 2.1 Energy absorption mechanisms

The unique macroscopic properties behind mechanical metamaterials emerge from the absorption mechanisms of their unit cell. Such unit cells can be formed by several structural elements, such as rods, beams and shell structures, and they can give distinct deformation modes when interacting with an external stimuli.

**Figure 2:**
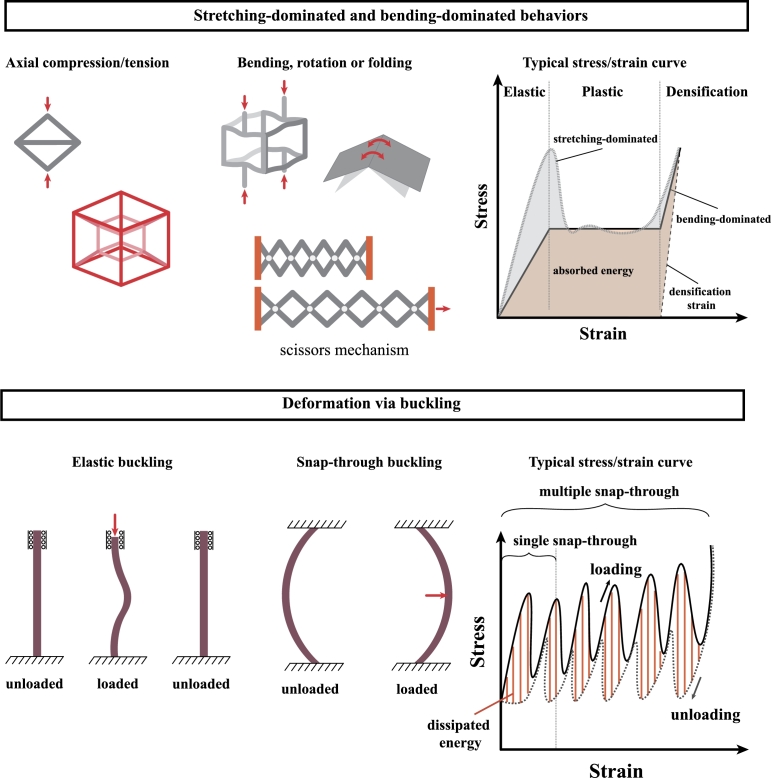
Main deformation mechanisms observed in mechanical metamaterials for absorbing energy.

Fig. 2 summarizes the main deformation modes related to the energy absorption ability of mechanical metamaterials. Those can be either stretching-dominated, bending-dominated, buckling deformation or a combination of such mechanisms, particularly when designing hierarchical materials. Stretching-dominated unit cells deform through uniaxial tension or compression of their inner elements, while bending-dominated ones deform by bending of their elements. Because of that, stretching-dominated unit cells can exhibit higher stiffness-to-weight ratio in comparison to their bending-dominated counterparts, being the former practical for dealing with loads in structural applications and the latter suitable for developing compliant structures [6], [17], [18]. For instance, microlattice materials formed by octet-truss unit cells exhibited a linear scaling relationship of $E/E_{s}∼\rho /\rho_{s}$, unlike those made of bending-dominated unit cells, which relationship is $E/E_{s}∼{\left(\rho /\rho_{s}\right)}^{2}$. Here, $E/E_{s}$ is the ratio of the Young's modulus of the macroscopic structure and the Young's modulus of the material, while $\rho /\rho_{s}$ is the relative density defined by the ratio of the macroscopic density and the density of the basis material. This means that stretching-dominated unit cells can exhibit high strength with a lower relative density in comparison to the bending-dominated counterparts. Even though stretching-dominated unit cells exhibit improved strength density ratio, local buckling may occur under large compressive strains as well as high stress concentration since the unit cell elements cannot rotate or bend due to the high nodal connectivity. In contrary, bending-dominated ones are compliant and lightweight, and their internal elements can rotate, fold or bend reducing the risks of buckling. To combine the benefits of both stretching- and bending-dominated unit cells, hybrid lattice structures [19], [20] and multiscale hierarchical structures [21] have been developed. The combined elements in hybrid structures have similar scale, while in hierarchical structures, the scale varies. Although such structures demonstrated enhanced properties, local buckling can still occur, preventing their reuse. Composite bending-dominated lattices were later developed to suppress buckling while maintaining high strength weight ratio and good cyclic performance [22]. To reduce the stress concentration, smoothness was introduced in the connections of shell lattices, which resulted in improved strength and energy absorption capacity [23], [24].

Other deformation mechanisms are reached through motion structures or deployable structures, from which their shape can be reconfigured and changed through folding and unfolding to the cost of zero (or near zero) deformation energy [25]. Such folding motion has been achieved by rigid deployable structures that consist of one or multiple degrees of freedom kinematic chains made of rigid structural elements connected by joints. Some examples are truss structures containing scissor joints, which expand and collapse their shapes, and plate structures connected by hinges at their edges. In addition to that, flexibility can be embedded to deployable structures, which gives flexile elements that under external constraints are folded in a small volume, storing potential energy, as commonly observed in automotive airbags and shield for space telescopes. Deployable structures formed by rigid and flexible elements have been also proposed to provide high stiffness, high flexibility, and lightness. Examples of such structures are compliant and pantographic mechanisms–rigid elements connected by flexible joints– and tensegrity structures–rigid elements connected by flexible cables.

Although elastic and plastic buckling are often considered as an undesirable mechanical instability, many mechanical metamaterials are designed to possess such mechanisms tailored in such a way that energy absorption is enhanced and the metamaterial can be recoverable and reusable. In fact, incorporating buckling can result in geometrical nonlinearities–while the constituent materials could still be in the linear regime–, converted into mechanical bistability [26], [27], multistability [28], [29], or programmable behavior [30], [31]. Snap-through behavior is a common case of bistability, where a state shifts quickly to the second state when the load applied to structures exceeds the critical load, as illustrated in Fig. 2. During the transition stage, the initial positive stiffness converts to negative stiffness during deformation and returns to positive stiffness as second state is reached. When looking to the energy involved in such process, the structure absorbs energy until an unstable point and it releases energy when changing states. This gives the ability of snap-through structures in absorbing-energy and reusable properties. Finally, bistable structures can be combined into multistable structures that can provide different stable configurations with a vast variety of deformation modes, enhancing their energy absorption properties.

### 2.2 Energy absorption performance under diverse loading conditions

When defining the material and the mechanism for absorbing energy in a mechanical metamaterial, it is important first to investigate the nature of the load at which the metamaterial is subjected to. Loading conditions can be described by their velocity, which can be classified as quasi-static, low-velocity impacts, and high-velocity impacts. This results in structural responses with distinct strain rates, implying the relevance of considering inertial effects and wave propagation [32].

Quasi-static loads cause relatively low strain rates ($10^{-5}∼10\;s^{-1}$) and inertia effects are negligible, which results in homogeneous deformation mode. In this mode, the deformation and collapse occurs at the weakest point of the structure [33]. In cellular structures formed by struts, the nodes are the weakest point where high stress concentration occurs. For instance, when chiral structures buckle, the nodes rotate, resulting in large local strains that brings to a structural failure [34]. The energy absorption performance of structures under quasi-static loads is attributed to such localized events.

In case of dynamic loads, cellular structures can exhibit different deformation modes due to the strain-rate and inertia effects [35]. In high-velocity loading conditions (strain rates in the range $10^{3}∼10^{5}\;s^{-1}$), shock modes are identified, where transverse crushing of the structure is observed at the impact front, independently of the weakest structural point [36]. Structures under low-velocity loading conditions, where strain rates with range $10∼10^{3}\;s^{-1}$ are observed, experience transition modes, where homogeneous and shock modes can be present. For instance, regular honeycombs under low-impact velocity show double V-shaped bands near the crushing edge and at the support edge. Auxetic sandwich panels under blast loading have also indicated improved energy absorption in comparison to traditional sandwich panels due to the material concentration at the impact zone attributed to the auxetic effect [37]. Fancher et al. (2023) [38] investigated the kinetic energy transmission performance in response to impact of an impactor with different mass and velocity conditions. They concluded that damping can affect significantly the performance, which can underperform a more traditional materials if the material and impact conditions are not properly selected. For high impact velocity, inertia effect becomes significant and the V-shaped bands disappear and I-shaped bands are identified at the crushing edge [39]. In case of auxetic structures under quasi-static and low-velocity loads, the crushing stress was related to the angle related to the auxetic effect. However, under high-velocity loads (strain rates in the range $10^{3}∼10^{5}\;s^{-1}$) the crushing stress was not affected by changing the angle [40]. Depending on the impact velocity, the energy absorption ability of mechanical metamaterials can be attributed to localized events, such as buckling and shear bands, and to global events, such as densification at the impact zone.

### 2.3 Unique properties of mechanical metamaterials

Based on the loading conditions and designed deformation mechanisms described earlier, mechanical metamaterials can exhibit their unique properties, which will be presented in this section.

#### 2.3.1 Auxetic metamaterials

Metamaterials possessing a negative value of Poisson's ratio, which are also named as auxetic metamaterials, have the interesting phenomenon of contracting (expanding) transversely under longitudinal compression (tension) load [41], [42]. From the classical elasticity theory, the Poisson's ratio is related to other mechanical properties – Young's modulus and shear modulus – by the expression $G=\frac{E}{2(1+\nu )}$. Therefore, when the Poisson's ratio approaches −1, an infinite value of shear modulus is obtained. This results in many intrinsic properties, such as indentation resistance [43], thermal shock resistance, and fracture toughness [44]. Other works have also reported high shear resistance [45] and energy absorption [14].

**Figure 3:**
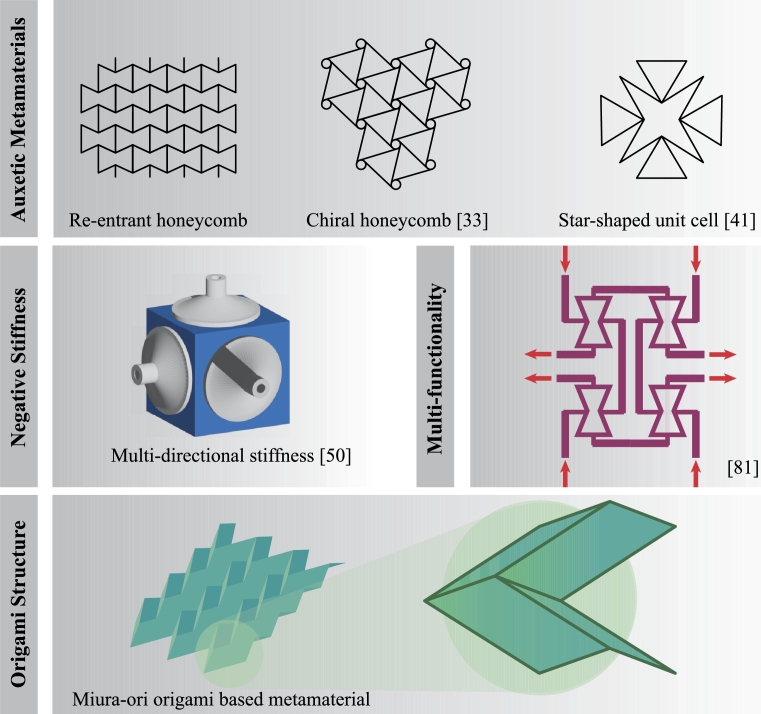
Examples of mechanical metamaterials for energy absorption application.

Such auxetic behavior originates from the deformation mechanisms exhibited by the microstructure under uniaxial loading. The most common microstructure geometries are the re-entrant structures and chiral structures, as shown in Fig. 3. The deformation mechanism of re-entrant structures [46], [47] consists of mainly folding of their structural elements at the joints. The auxetic effect is tuned by adjusting the geometric parameters of the structure, such as the strut thickness, the length ratio, and the re-entrant angle. In chiral structures, the deformation mechanism is governed by the nodes rotation and cell elements flexure when the structures are under tensile/compressive loadings. For example, chiral honeycomb structures [48], [49], [50] achieve this effect through (un)wrapping of the ligaments around the circular nodes under external loads, while rib flexure and/or hinging is observed in star honeycomb structures [51]. Subsequent research has explored new structures to enhance the mechanical properties and energy absorption of auxetic metamaterials. This involves investigating different ligament geometries [52], [53], [54] and incorporating additional structural elements into existing re-entrant structures [55], leading to improved auxetic properties.

When analyzing auxetic structures under various loading speeds, it has been observed improvements of energy absorption in re-entrant honeycomb structures in comparison to regular hexagonal honeycomb due to the early densification. However, under high peak stress, the re-entrant structure may generate higher stress to dissipate amount of impact energy [56]. Although many studies presented the effect of energy absorption capacity of auxetic structures, a comprehensive understanding of the relation between the deformation mechanism and macrostructure properties under dynamic loads–especially high-strain rates–is still required.

#### 2.3.2 Negative stiffness

Metamaterials can also exhibit unique properties through structural instabilities. One such example is negative stiffness (NS) metamaterials, characterized as recoverable structures due to their energy absorption relying on elastic deformation of internal structural elements. Typically, negative stiffness metamaterials consist of axially constrained curved beams with snap-through behavior [57], where displacement increases as the force increases until the beams shift from one stable position to another followed by a force level reduction, from which the stored strain energy is released. These beams can be arranged in various configurations, including honeycomb [58], [59], cubic [60], and hexagonal structures [57]. Other structures capable of achieving negative stiffness behavior include disk structures [61], [62], sleeve-type structures [63], [64], among others.

The buckling effect observed in NS metamaterials has been explored in impact absorbers. The early models of NS metamaterials primarily absorbed energy in one direction and could withstand tensile or compressive loads in a single principal direction. Recent research has concentrated on enhancing NS metamaterial features by expanding their energy-absorbing capacity to multiple directions [60], [65] as shown in Fig. 3, their ability of bidirectional buffering and absorbing energy [66], and their adaptability for specific engineering applications [67], [68].

Although improvements on energy absorption capacity have been extensively explored, mostly under controlled loading conditions, an in-depth investigation of the mechanical response of NS metamaterials under various loading conditions is necessary. A computational study on the dependence of impact mass and velocity on energy absorption capacity has recently shown that the performance of bistable metamaterials can range from worse to superior, especially when damping is introduced in the model [38]. When dealing with high-strain rate impact problems, material behavior can be crucial since the constituent material of the bistable elements may undergo plasticity and fracture, which could rather decrease the efficiency of the bistable mechanism. To solve such impact problems, real-time tunability of mechanical responses of NS metamaterials could be potentially used, since it allows better control of the deformation stages [69].

#### 2.3.3 Exotic properties of foldable structures: origami, tensegrity, and pantographic metamaterials

Origami is a Japanese art of creating 3D objects from folding a paper sheet. This process creates patterns of flexible (crease) and stiff areas (thin panel), which usually results in two stable stages, named as folding and expanding stages. By controlling the number, order and orientation of folds, origami has served as inspiration for designing novel mechanical metamaterials possessing tunable stiffness, multi-stability and auxetic properties [70]. The initial studies on origami-based mechanical metamaterials were based on rigid origami, where the panels remain rigid during folding/unfolding and the crease deforms. Therefore, the strain energy is only related to the crease deformation. Miura-ori and Ron Resch patterns, for instance, have demonstrated negative Poisson's ratio and unusual load bearing capacity, respectively [71], [72], [73]. By changing the geometry of the crease and/or panel, the mechanical properties of such rigid origami can be tuned. Although rigid origami has a simple pattern of deformation, its application can be limited. To extend the applicability of origami-based mechanical metamaterials, works have included the flexibility of origami panels, which add the contribution of strain energy of the panel deformation. Zhai et al. (2020) [74] proposed curved origami panels to achieve in situ stiffness manipulation, which could be positive, zero or negative. A detailed experimental validation of the intrinsic properties of an origami-based cellular structure was presented by Yamaguchi et al. (2023) [75]. In-depth analysis is still required to understand the mechanisms behind the exotic features of origami metamaterials [76].

Unlike origami structures, where deformation occurs mainly at the cease, pantographic metamaterials exhibit unique mechanical properties due to the pantographic motif, which is a mechanism characterized by a zero-energy extension/compression deformation mode. This kind of mode is commonly seen, for example, in scissor mechanisms, where two structural elements are connected by rotating or flexible joints [77]. Such metamaterials can undergo large deformations while maintaining their overall structural integrity, since their mechanism can enable effective load redistribution making them ideal for absorbing mechanical energy and conforming to complex surfaces, such as in smart textiles and wearable electronics [78]. 3D micro-metric pantographic structures have exhibited resilience against damage and different deformation patterns, due to their unique topology, where the recoverable deformation energy was mostly stored in the bending of the beams or in the torsion of the connecting elements [79]. Although the mechanical properties of pantographic metamaterials have been explored for cycling loads, the same is missing for impact waves with varied strain rates.

Tensegrity metamaterials differ from the previous foldable structures due to their unique internal load-transfer mechanism and deformation, which results from selfstressed framework formed by isolated compressive elements (bars) and tensile elements (cables). Under uniaxial compression tests, tensegrity metamaterials exhibited stiffening or softening response [80] and delocalized deformation mechanisms and a stable stress-strain response in comparison to octet architecture and Kelvin foam [81], which gives improved failure resistance and energy absorption in comparison to conventional lattice structures. However, the experimental realization of metamaterials of tensegrity is still in its early stages, particularly due to the complexity of assembling the network of bars and cables. To address this issue, have recently reported tensegrity-like structures, where the bars-cables framework is replaced by only bars framework connected to compliant hinges, giving a monolithic structure that can be fabricated by additive manufacturing techniques [82]. The only-bars framework has been also applied to the fabrication of micro-scale tensegrity structures through the AM technique of multiphoton lithography [83].

#### 2.3.4 Multi-functional mechanical metamaterial

Recent advancements in mechanical metamaterials for energy absorption have emphasized the design of structures with multi-functionalities, rendering them suitable for addressing more intricate engineering challenges. This section presents examples of current multi-functional mechanical metamaterials.

Hewage et al. (2016) [84] proposed a double-negative mechanical metamaterial, comprising an auxetic host embedded with negative stiffness elements. This design offers both negative stiffness and negative Poisson's ratio, potentially resulting in improved vibration damping. Inspired by the double negative design reported by Hewage et al. (2016) [84], other works have explored different 2D metamaterial designs displaying multiple negative properties [29], [85]. Some designs have demonstrated a strong coupling between two negative properties, which may affect the energy dissipation efficiency [29]. To overcome that, a realization of double negative in 3D metamaterials was presented by Pan et al. (2024) [86], where a less strong coupling between the negative mechanical properties was induced.

In their study, Zhang et al. (2021) [87] integrated re-entrant and chiral structures to investigate two deformation mechanisms: bending at the re-entrant scale and chirality. This integration led to enhancements in both in-plane effective stiffness and Poisson's ratio. Additionally, Yasuda et al. (2015) [73] proposed combining re-entrant structures with origami, demonstrating analitically and experimentally both negative Poisson's ratio and structural bistability. In terms of energy absorption for different load conditions, re-entrant circular honeycombs exploring structural hierarchy on the meso-scale and functional gradient on the macro-scale have shown improved performance in comparison to traditional re-entrant structures [88].

### 2.4 Application of MMMs in engineering problems

In this section, we outline the potential applications of MMM mechanisms for absorbing energy. Table 1 provides a summary of MMM designs featured in this review, categorized by property, their deformation mechanisms at unit cell level, potential applications, challenges and limitations related to the functionality, and scalability issues.

**Table 1:** Summary of MMM in terms of deformation mechanism at unit cell level. Their potential engineering applications are highlighted along with the challenges, limitations and scalability issues.

Feature	Deformation mechanism	Potential application	Challenges	Scalability issues
Auxetic behavior	Bending-dominated, stretching-dominated	1) Multi-axial energy absorption [51]	1) Energy absorption investigation restricted to compressive loads	1) Anisotropic behavior for printed structures, due to interlayer defects and pores [55]
	Chirality	2) Improved stiffness and auxetic behavior for larger deformations [55]	2) Energy absorption process relies on plastic deformation, reducing the use to a single application	

Negative stiffness	Elastic buckling due to geometry nonlinearity,	1) Multi-axial energy absorption with self-recovery snap-through response [60], [66]	1) Further investigation of energy absorption under distinct impact conditions	1) Study required for NS lattice structure with sufficient number of cells per side [60]
	Snap-through behavior	2) Energy absorbing feature by using cylindrical structures [67], [68]	2) Damping effects of functionality [67]	2) Numerical models cannot predict well asymmetric modes caused by manufacturing imperfections [67]
		3) Development of stretchable devices [68]		3) Boundary constraint alters stiffness of outer layers

Foldable structures	Crease folding and panel bending; scissor mechanism	1) In-situ stiffness manipulation and curved origami-based grippers [74]	1) Manually tunability of stiffness [74]	1) Connection of the panels [74]
		2) Automotive airbags, implants [75], smart textiles, and wearable electronics	2) Property evaluation in linear-elastic regime [75]	2) Planar configurations not suitable for three-dimensional construction

Multi- functional	Snap-through behavior	1) Shape reconfiguration and shock isolation [29]	1) Require investigation under different loading conditions	1) Amount of unit cells not enough for demonstrating scalability
		2) Impact protectors in aerospace, soft robotics [84], [85], [86], [89]	2) Large friction coefficient [86]	

While several studies have validated the MMM's mechanisms experimentally, most focus on prototypes developed for laboratory testing, often manufactured via 3D printing technology. When adapting such MMM designs to scales differing from the numerical and experimental studies, it is important to consider possible size effects. Such effects could be attributed to geometrical scaling, microstructural effect, anisotropy and non-linearity, boundary interface effects, and manufacturing and defect sensitivity. By investigating these effects, MMMs can be applied to their corresponding potential applications.

## 3 Vibration control through acoustic/elastic metamaterials

Acoustic/elastic metamaterials can control vibrations by means of their frequency-dependent effective mechanical properties. At specific frequency ranges, the propagating wave interacts with the AEMM resonant inclusions, causing them to resonate in a certain vibration mode associated to the frequency-dependent mechanical property. The first work demonstrating the vibration control through a resonant mechanism was proposed by Liu et al. [90]. In their work, a composite containing locally resonant structural units showed negative effective mass density (EMD) at frequency ranges near the microstructure resonance. The negative EMD is associated to the out-of-phase translation motion between the main structure and its resonant element, which is called dipolar resonance [91]. Similarly to the EMD, negative values of effective bulk modulus (EBM) [92] and effective shear modulus (ESM) [93] have been also obtained due to monopolar and quadrupolar resonant behaviors of the MM microstructure, respectively. Some works have also reported interesting mechanical properties by specific spring arrangements, as observed in structures possessing effective Young's modulus (EYM) [94]. To create ultra-low band gap frequencies by using resonator arrangements, a system formed by heavy masses and low stiffness spring would be required, which could result in unpractical designs. To overcome such limitation, quasi-zero stiffness (QZS) and inertial amplifications (IA) designs have been proposed to control the effective stiffness and effective mass, respectively. In this section, a brief description of each mechanical property is presented. Spring-mass systems are used to illustrate the mechanism behind the negative phenomenon. Then, special metamaterial designs possessing multiple negative properties are presented.

### 3.1 Effective negative properties

**Figure 4:**
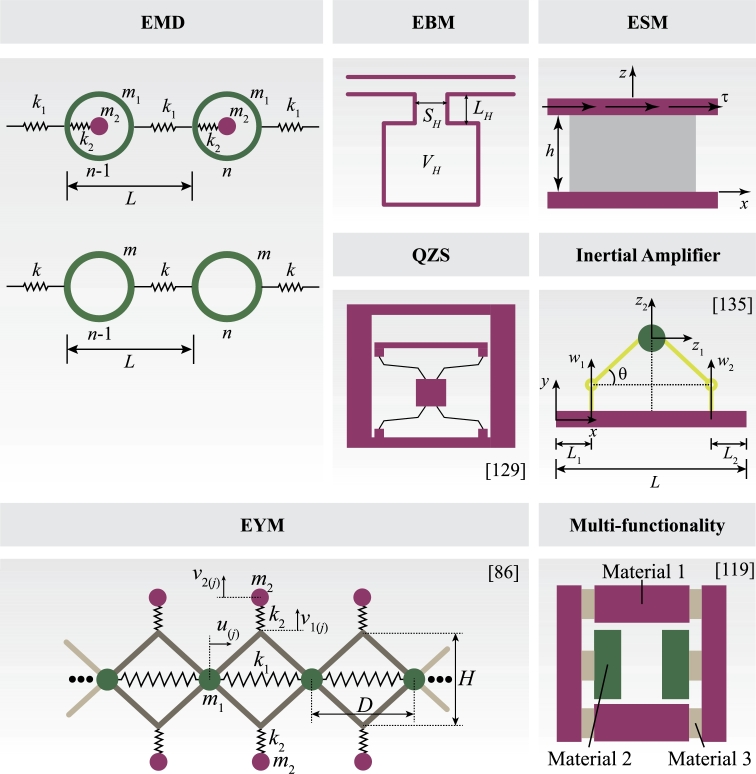
Examples of mechanical metamaterials possessing effective properties for controlling vibrations.

Inspired by the initial evidence of exotic mechanical properties [90], [95], research on negative EMD was pioneered by [96], [97], [98]. This property was first theoretically evaluated using a mass-in-mass system connected by springs [99], as illustrated in Fig. 4.

**Figure 5:**
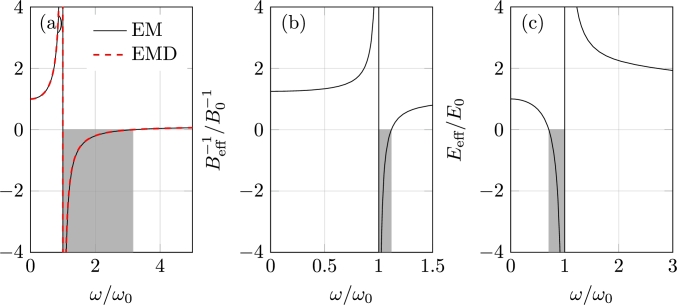
(a) Nondimensional effective mass *m*_eff_/*m*_st_ and mass density *ρ*_eff_/*ρ*_st_ (left); (b) nondimensional effective bulk modulus $B_{\text{eff}}^{-1}/B_{0}^{-1}$ (center); and, (c) nondimensional effective Young's modulus *E*_eff_/*E*_0_ (right). The gray rectangle indicates the region with negative values of the respective effective properties.

The dynamic behavior of such mass-in-mass systems was further investigated through effective systems whose effective mass $m_{\text{eff}}$ is given as

(1) $$m_{\text{eff}}=m_{1}+\frac{m_{2}\omega_{0}^{2}}{\omega_{0}^{2}-\omega^{2}},$$

where $\omega_{0}=\sqrt{k_{2}/m_{2}}$ is the local resonance frequency related to the mass $m_{2}$. Fig. 5a shows the variation of the effective mass over frequency for a mass-in-mass system with properties $m_{1}=1$, $m_{2}=9$, $k_{1}=1$, $k_{2}=0.1$, and L=1. It is worth noting that when the frequency approaches the resonance of the inclusion ($\omega /\omega_{0}=1$), the effective mass becomes negative. Physically, this means that the acceleration of the effective mass is opposite to the direction of the applied excitation, hence the response amplitude is reduced.

When modeling the equivalent monoatomic system as a elastic solid, the effective mass density is expressed as

(2) $$\frac{\rho_{\text{eff}}}{\rho_{s}}=\frac{\theta }{\delta (1+\theta ){\left(\omega /\omega_{0}\right)}^{2}}{\left\{\cos^{-1}\left\{1-\frac{\delta }{2\theta }\frac{{\left(\omega /\omega_{0}\right)}^{2}[{\left(\omega /\omega_{0}\right)}^{2}-(1+\theta )]}{{\left(\omega /\omega_{0}\right)}^{2}-1}\right\}\right\}}^{2},$$

where $\theta =m_{2}/m_{1}$, $\delta =k_{2}/k_{1}$, $\omega_{0}^{2}=k_{2}/m_{2}$, and the static mass density $\rho_{s}=(m_{1}+m_{2})/L$. Fig. 5a shows the variation of effective mass density as a function of frequency for the same mass-in-mass system's features. It is also noticed the presence of a negative region at a frequency range nearby the resonance of the mass-in-mass system, which is characterized by the out-of-phase motion of the system with respect to the excitation. Since the longitudinal wave speed is given by $c_{L}=\sqrt{E/\rho }$, a negative value of *ρ* results in a complex wave speed, which physically means that the wave attenuates.

Building upon findings from simple discrete models, several continuum designs have been developed to attain the EMD. These designs employ various approaches, including impedance mismatch by pairing a stiff core with a soft material [100] and spring-mass systems [101]. Alternatively, single-phase unit cells are explored to achieve EMD through the microstructure topology of the unit cell [102], [103]. External resonators, such as metamaterial plates formed by resonant pillars [104], [105], have also been utilized to create EMD, particularly for waveguiding and confinement applications. Anisotropic EMD MMs, which contain independent EMD values for each propagation direction, have been investigated for converting wave types [106] and mitigating waves with different orientations [107]. While metamaterials possessing negative EMD have made significant contributions to wave attenuation, the limited range of negative EMD in specific designs, as depicted in Fig. 5), may constrain their applicability to problems involving waves with multiple frequencies, for instance, impact loads. To address this constraint, strategies such as grading resonant structures and exploring parallel arrangements of resonators have been proposed to broaden the attenuation bandwidth [100], [102], [103], [108].

Elastic materials exhibit a positive bulk modulus, representing their resistance to compression/expansion. In case of local resonant metamaterials, the interaction between propagating wave and resonant subwavelength elements can yield a frequency-dependent effective bulk modulus. Fang et al. (2006) [92] initially observed negative EBM in an acoustic metamaterial composed of Helmholtz resonators, leading to out-of-phase bulk mode propagation. Considering a single Helmholtz resonator of volume $V_{H}$, neck size $S_{H}$ and effective length $L_{H}$, as illustrated in Fig. 4b, the EBM $B_{\text{eff}}$ is defined as [109]

(3) $$B_{\text{eff}}^{-1}=B_{0}^{-1}\left(1+\frac{\omega_{1}^{2}-\omega_{0}^{2}}{\omega_{0}^{2}-\omega^{2}}\right),$$

where $\omega_{1}=\omega_{0}\sqrt{\left(1+V_{H}/V\right)}$ and $\omega_{0}$ is the resonant frequency of the Helmholtz cavity defined as

(4) $$\omega_{0}=\sqrt{\frac{B_{0}S_{H}}{V_{H}\rho_{0}L_{H}}},$$

being $B_{0}$ and $\rho_{0}$ the bulk modulus and density of the air in the tube. Similarly to the negative EMD, the EBM can be also explained by a spring-mass system, where the fluid in the neck works as an effective oscillating mass and the fluid in the chamber body acts as an effective spring. As illustrated in Fig. 5b, the EBM becomes negative close to the resonance.

While EBM has predominantly been studied in acoustic contexts, some studies reported elastic metamaterials engineered to achieve negative bulk modulus through rotational resonance of the unit cell. EBM in elastic metamaterials has contributed significantly to wave filtering applications [110], [111], [112].

The negative EYM was theoretically proposed by Huang and Sun. (2011) [94]. Their model consists of a resonator chain as presented in Fig. 4, where a spring with stiffness $k_{1}$ and length *D* is connected to four rigid rods whose mass were concentrated at the mass $m_{1}$. The perpendicular responses are given by two sets of mass-spring systems with elastic constant $k_{2}$ and mass $m_{2}$ connected to the rods. The effective Young's modulus of this system is given by

(5) $$\frac{E_{\text{eff}}}{E_{0}}=\frac{\delta }{\theta }\eta^{2}{\left\{\cos^{-1}\left[1-\frac{(\eta^{2}-1)\eta^{2}}{2[(\theta /\delta +\theta \mu /2)\eta^{2}-\theta /\delta ]}\right]\right\}}^{-2},$$

where $\theta =m_{2}/m_{1}$, $\delta =k_{2}/k_{1}$, $\mu ={\left(D/H\right)}^{2}$, and $\eta =\omega /\omega_{0}$. Fig. 5c illustrates the relation between the nondimensional frequency and the EYM for a lattice structure with properties θ=2, δ=0.5, and μ=4, where a negative EYM is also observed close to the resonance.

Inspired by the effective Young's modulus model proposed by Huang and Sun (2011) [94], new MMs have been developed to suppress the propagation of longitudinal [113] and flexural waves [114] in continuum structures by transforming the direction of wave propagation, which have been fundamental for controlling vibration in common engineering structures, such as beams and plates.

Different from acoustic metamaterials, from which the acoustic medium is defined by the effective mass density and bulk modulus, the elastic counterparts have an additional parameter, the effective shear modulus (ESM), since the solid medium supports both longitudinal and transversal waves. As the negativity of the effective mass density and bulk modulus was result of an anti-parallel motion between the inclusions and the matrix, the similar behavior occurs for negative shear modulus MMs. Fig. 4 shows an elastic material under a shear test where the bottom surface is fixed and a shear stress *τ* acts on the top surface, considering an infinite long elastic material, the shear modulus $\mu_{\text{eff}}$ can be expressed as

(6) $$\mu_{\text{eff}}=\tau \frac{z}{u_{x}(z)}=\tau \frac{h}{U_{x}},$$

where $u_{x}(z)$ is the displacement of a material layer at *z* along the *x* direction, and $U_{x}=u_{x}(h)$ is the top surface displacement. As observed in Eq. (6), a negative shear modulus is obtained when the direction of the displacement field of the material layer at *z* is anti-parallel to the direction of the applied shear stress. When it comes to dynamics, some works have derived the effective shear modulus based on effective medium and analytical models [93], [115], [116]. The property of negative shear modulus can be widely explored as selective wave filtering. For instance, Wang et al. (2020) [117] has demonstrated the control of shear waves by exploring the torsional resonance of an elastic metamaterial plate with a stub on one side.

### 3.2 AEMMs with multiple negativity

Researchers have delved into understanding various resonant mechanisms to combine effective properties within the same frequency range, leading to a negative refractive index. This results in an anti-parallel phase and group velocities. The initial designs of MMs showcasing double negativity involved a combination of solid and fluid media [91], [118], [119]. However, these designs were complex to manufacture, prompting the development of double-negative metamaterials using only solid materials (Fig. 4). This was initially performed by developing theoretical models [120], followed by proposals for more compact designs aiming to achieve simultaneous negative effective mass density and negative bulk modulus through chiral structures [110], [121], [122]. Such structures induce rotational and translational resonance, thereby producing negative effective bulk modulus and negative effective mass density at specific frequencies. Different unit cells have been suggested to independently regulate the negative mass density and the negative modulus [108], [111], [112], [123]. Due to the strong coupling among different deformation modes in elastic medium, the independent tuning of density and stiffness modulus becomes more complex. To address such issue, Oh et al. (2016) [124] introduced an elastic metamaterial made of two independent resonators each of which realizing negative density and stiffness.

In elastic MMs, achieving double negativity also occurs when both the mass density and shear modulus are negative within specific frequency ranges [117], [125], [126], leading to dipolar and quadrupolar resonances. Thus, a negative transverse wave velocity is obtained, i.e. $c_{t}=\sqrt{\mu }(\sqrt{1/\rho })<0$, implying a negative refraction. This property could be used for mode conversion from transversal waves to longitudinal waves. Further studies also reported triple negativity – negative EMD, EBM, and ESM – at the same frequency range [127], which provides various dynamic features [128], such as fluid-like behavior and negative phase velocity for longitudinal and transversal waves. Therefore, having control of the effective parameters of elastic metamaterials enables the control of hybrid waves by means of polarized band gaps [129].

### 3.3 Quasi-zero stiffness

Previously discussed negative effective properties arose from simple mass-spring systems, resulting in low-frequency band gaps. However, achieving ultra-low band gaps in such systems required a heavy mass connected to a low-stiffness spring, which poses impractical design challenges. To address this, researchers have explored resonator designs with extremely low stiffness while maintaining structural strength. Some studies have introduced high-static-low-dynamic-stiffness resonators, generating band gaps at very low frequencies [130], [131]. The high-static-low-dynamic-stiffness property involves a negative stiffness mechanism counteracted by a positive stiffness system. While previous designs employed spring-mass systems to achieve high-static-low-dynamic-stiffness [130], [131], [132], recent research by Cai et al. (2020) [133] investigated negative stiffness systems using compact bistable elastic structures. Additionally, compact QZS metamaterials have been developed using resonators comprising programmable curved beams [89], [134], magnet rings and coil springs [135], [136], and parallel multi-segment beam systems [137], [138], [139] (Fig. 4).

### 3.4 Inertial amplifiers

Another approach to reduce the band gap involves amplifying the effective mass of a component through displacement amplification mechanisms, also known as IA (Fig. 4g). The amplified motion generates enhanced inertial forces, thereby elevating the effective mass of the system and consequently lowering the resonance frequency. Discrete models have been utilized to elucidate this mechanism in both infinite [140] and finite media [141]. Building upon this concept, an extension to continuum models was proposed by [142], [143], [144]. Another mechanism of inertia amplification involves employing chiral links to couple the axial and rotational motion of metastructure elements, resulting in an additional inertia contribution without altering the total mass [145], [146]. Further chiral inertial amplification structures with distinct unit cell arrangements were investigated, from which syndiotactic arrangement exhibits ultra-broad low-frequency band gap, although still limited by the stiffness and mass of the structure [147]. Novel chiral structures are being investigated to break the mass and low-frequency band gap relation [148].

### 3.5 Application of AEMMs in engineering problems

In this section, we explore the practical applications of the numerical and experimental analysis of wave control mechanisms in AEMMs. Table 2 categorizes AEMM models and prototypes, similar to the previous section. Notably, experimental validation is limited, primarily focused on small-scale unit cell designs, while numerical investigations have tackled larger engineering problems. The main challenges include managing geometry complexity, tuning dynamic properties, and addressing nonlinearities, which are crucial considerations for future mechanical metamaterial designs.

**Table 2:** Summary of AEMMs in terms of desired dynamic property. Their typical band gap principles are highlighted along with the potential engineering applications, challenges, limitations and scalability issues.

Feature	Band gap principle	Potential application	Challenges	Scalability issues
EMD	nonlinearity due to geometric change [101], flexural mode excitation [100], [102], [103], axial and shear rod deformation [103]	vibration attenuation at quasi-static frequency [101], nondestructive evaluation and structural health monitoring [105], wave redirection mechanisms [107], seismic shields [149]	Material damping effect on attenuation values [102],	Friction effect for small-scale nonlinear metamaterial [101], perfect interface coupling assumed in numerical models
EYM	Inner resonance due to stiffness contrast [113], interaction between four-link mechanism and lateral resonators [114]	Device for controlling the direction of transmitted elastic waves [113]; wave conversion [114]	Damping effect reflecting on resonance performance [114]	Principle of wave control based on homogenization method, therefore wave length should be larger than unit cell size [113]
QZS	Energy transfer from metamaterial to energy source [136], combination of negative- and positive-stiffness structure [133], large deformation of compliant structure [137]	Ultralow vibration isolation (lower than 20 Hz) [136], [139]	Damping effect reduces wave attenuation [139]; enhanced stiffness nonlinearity can degrade wave attenuation [133], [137]; manufacturing imperfections shift band gap [139]	Band gap is only broadened when scaling up the mass and stiffness of the resonator [138]
IA	Increase of effective inertia by generating rotational motion from axial motion [145], [146]	Isolate seismic surface waves [144]	Band gap induced by IA is quite narrow [144]; geometry relies on thin structures, requiring stress evaluation [146]	Geometry of the ligaments should be changed to shift the stop band, which may induce different behaviors for distinct scales [146]
Double	translational motion of the resonator [108], [123], combination of torsional, bending and compressional resonance [117]	Polarization filtering, abnormal refraction, and mode conversion (elastic switches and seismic waves control) [117], [121], [122], [124]; structural protection [150]	Broad band gap induced by inclusion of more resonators, increasing geometry complexity; complex coupling of longitudinal and shear waves in 2D and 3D problems	Sensitivity of negative refraction to imperfections can be significant when cell size approaches the wavelength of propagating wave [121]; boundary and material inhomogeneity effects on band gap prediction

## 4 Future direction of mechanical metamaterials: hybrid metamaterials for simultaneous vibration control and energy absorption

The demand for single integrated metamaterials that exhibit multiple functionalities allowing improved mechanical performance and vibration control is increasing nowadays to solve complex engineering problems. With the advances in computational science (optimization schemes [151] and inverse design by artificial intelligence) and additive manufacturing technology, multi-functional metamaterials, termed as hybrid mechanical metamaterials, are being developed to attain the static and dynamic properties discussed in the previous sections. To understand the development of HMMMs, this section summarizes the most recent design processes employed for obtaining the multi-functionality of HMMMs. We highlight the limitations of such design processes and potential solutions to overcome them. Finally, the potential application of HMMMs in real-world problems is discussed.

### 4.1 Design processes of hybrid mechanical metamaterials

**Figure 6:**
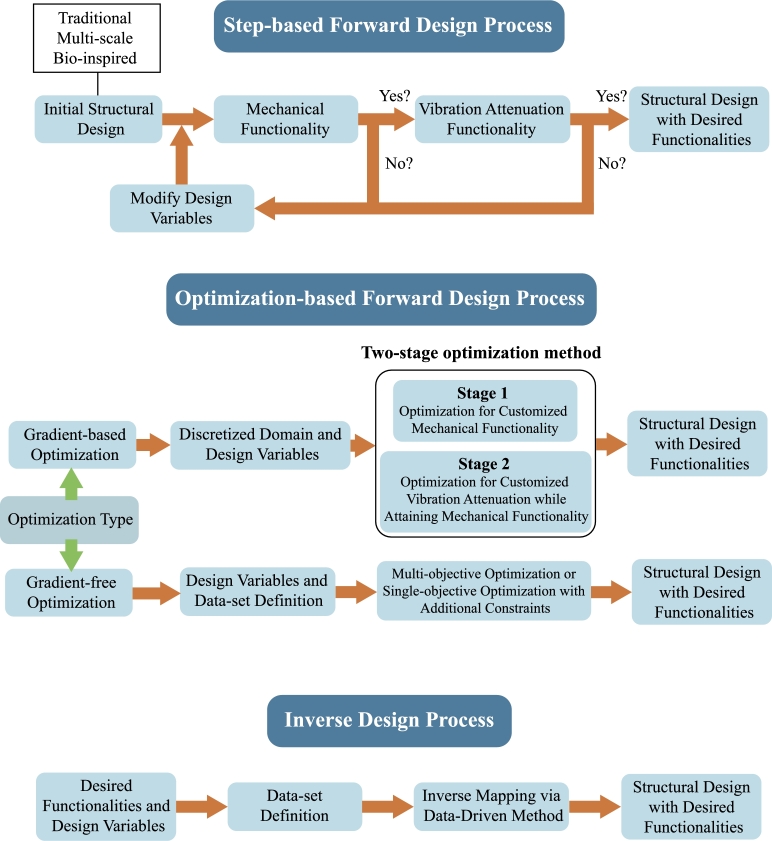
Design processes of hybrid mechanical metamaterials.

The multi-functionality of HMMMs can be attained by two main design processes: the forward and the inverse design. The steps of each process are indicated in Fig. 6.

#### 4.1.1 Forward design process

The forward design of HMMMs has been performed by two approaches: step-based and optimization-based approach. In the first, a structural design and its design variables are initially defined. The structural design is commonly chosen from traditional, multi-scale and bio-inspired designs, which will be described later. Numerical or experimental simulations are performed to quantify each functionality. In case one of the desired functionalities is not achieved, the structural design is changed by modifying its design variables from which new studies are performed. This process repeats until a structural design with the desired functionalities is obtained.

In step-based forward design, the initial structural design often uses traditional geometry, focusing on evaluating the dynamic or static properties of metamaterials tailored for their respective purposes. The performance of traditional mechanical metamaterials were initially focused on their ability to support quasi-static, low-velocity, and high-velocity impact loads, as we highlighted to be dependent on their deformation modes. However, it was just recently that studies were performed to relate the deformation process with the vibration characteristics of the metamaterial. Some works have shown that band gap tuning can be achieved by the different deformation states of NS metamaterials [34], [152], [153], [154]. Chirality has been used to induce tunable phonon band gaps in 3D micropolar metamaterials [155]. The development of dynamic mechanical properties based on auxetic metamaterials has been possible by investigating the deformation modes of their unique topology – the local rotation of nodes and bending of ligaments can produce wave mitigation features. For instance, Chen et al. (2017) [156] showed that deformation modes of lattice metamaterials formed by sinusoidal-shaped beams with negative Poisson's effect result in tuned band gaps. Both properties were attributed to the sinusoidal shape of the lattice beams. Tao et al. (2022) [157] explores the tunability of acoustic resonant band gaps via the auxetic behavior of a negative Poisson's ratio structure. It was shown that by changing the compressive strain the width and position of the band gap is shifted. In other applications, band gaps within specific frequency ranges must be preserved under deformation. Such response has been achieved in micro-scale mechanical metamaterials exhibiting two distinct configurations and positive and negative Poisson's ratio [158]. Band gap tunability has been also observed in foldable metamaterials. Xu et al. (2021) [159] presented an origami-based solution for controlling torsional vibration in beams, employing a bistable resonator made of Kresling-pattern origami. The compression-torsional coupling behavior of Kresling origami was leveraged to create torsional band gaps. The same coupling behavior has been used in a graded origami-inspired structure made of beams and plates, whose dynamic features involve programmable, directional wave control [160]. Furthermore, a metamaterial consisting of various Kresling origami modules, leading to different deformation stages, has been designed [161]. The several deformation stages were able to open band gap in different frequency ranges, which exemplify the tunability of such structures. Static compression of tensegrity-inspired lattices structures also induces band gap tunability [162], [163], while the same ability has been explored by varying geometric parameters of flexible hinges in pantographic metamaterials [164]. In these studies, the metamaterial is subjected to compressive static loads, from which the deformation stages are obtained for different strain values. The dispersion characteristics of the metamaterial at each deformation stage are obtained, which usually give band gaps at distinct frequency ranges. This gives the metamaterial the ability of tuning band gaps when under static loads, while the same ability for larger strain rates still needs to be explored.

Multi-scale (hierarchical) approaches have been also used as initial structural designs, from which each functionality is attributed to structures of different scales. Zhu et al. (2014) [165] reported a chiral-lattice-based mechanical metamaterial beam embedded with multiple local resonators to achieve broadband vibration attenuation while keeping its load-bearing capacity. Qi et al. (2019) [166] proposed a hybrid latticed formed by a re-entrant and an anti-chiral structure. The vibration attenuation was realized by adding rubber coated mass inclusions into the local nodes of the hybrid lattice. Other nodal shapes resulted in ultralight lattice structures possessing negative Poisson's ratio and vibration control performance [167]. Guo et al. (2022) [168] proposed a lattice truss core sandwich metastructure exhibiting coupled Bragg scattering and local resonance mechanism. Since the resonator periodicity is different from that of the host lattice structure, the metastructure can be modeled as a multiscale structure. The analysis of such structure is performed via a multiscale modeling method based on the combination of the dynamic homogenization method and the spectral element method. The metastructure achieved a wide low-frequency band gap using less local resonators compared with traditional metastructures, which gives a high load-bearing capacity. As an alternative for the high additional mass ratio requirement for low-frequency attenuation, the mass component in the macroscopic metamaterial is replaced by microscopic particle materials. The multi-scale design showed broader attenuation while retaining the lightweight feature [169]. In terms of enhancing energy absorption, Zhou et al. (2022) [170] performed impact testings with distinct load conditions on re-entrant structures embedded with local resonators. The experimental results showed that the impact mitigation capacity of the local resonant re-entrant structure was approximately 22.6% better than the conventional re-entrant structure. Dual-functional hierarchical mechanical metamaterials with vibration insulation and energy absorption features for quasi-static compression was proposed by Zhang et al. (2022) [171]. Recently, Zheng et al. (2024) [172] investigated the energy absorption performance of a multi-resonator honeycomb metamaterial, where a reduction of 80% of reaction force due to a blast load was observed, although crushing stress levels have not been reported. Hierarchical metamaterials have also been developed to attain band gap tunability. For instance, small re-entrant unit cells are added to the nodes of re-entrant structures to obtain a tunable band gap. The band gaps were attributed to the deformation mode of the small re-entrant designs, while tunability was achieved by applying compressive (tensile) strain to the overall structure [173]. In the same direction, Ma et al. (2023) [174] added square unit cells with mass inclusion to the nodes of the re-entrant structure. The band gap tunability was explored by changing the sizes of the resonators. Experimental studies were conducted in hexachiral local resonant metamaterial to demonstrate improved mechanical and vibration attenuation performance by exploring the deformation modes of the chiral frame [175]. In foldable metamaterials, Jiang et al. (2023) [176] proposed a tunable hybrid origami metamaterial by integrating local resonators into traditional Miura origami panels. The proposed metamaterial demonstrated improved wave mitigation capabilities, with a 25.7% reduction in reaction peaks compared to traditional Miura origami structures.

The unique characteristics exhibited by biological structures provide insights for designing novel mechanical metamaterials [177]. Several works have proposed energy-absorbing materials and structures inspired by animal bones, shells, and wings or leaves of plants and mushroom gills [178], [179]. To account for both impact energy absorption and impact-induced stress wave attenuation, composites inspired by the beak of woodpeckers were designed, where distributed soft inclusions were embedded in hard matrices [180]. Nacre-like materials have shown enhanced mechanical properties due to an alternating pattern of soft protein layers and stiff aragonite tablets [181]. An extended analysis of their vibrational response was performed, revealing that the incorporating hollow platelets together with the application of specific prestress states result in improved wave absorption capability. Tensegrity metamaterials resembling biological tissues have demonstrated tailored static and dynamic properties by tuning the topology, geometry, and induced prestress [182].

While the step-based forward design is straightforward, it often requires multiple iterations to achieve the desired functionalities. This process becomes increasingly time-consuming when additional functionalities are introduced or when handling complex 3D geometries with numerous design variables. To address these challenges, topology optimization has been integrated into the forward design stage, enabling systematic identification of the optimal material distribution for maximum performance within design constraints [183].

The optimization process has been performed via gradient-informed and gradient-free methods, as indicated in Fig. 6. Gradient-informed methods use derivatives of the objective function with respect to the design variables to evaluate how small changes in the design variables affect the objective function and constraints. Solid isotropic material with penalization [184], level set [185], and evolutionary structural optimization [186] is some examples of gradient-based methods that have been applied for optimizing metamaterial designs to provide improved static or dynamic properties. Simultaneously optimizing metamaterials with static and dynamic properties increases the complexity of the problem, necessitating the exploration of new optimization methodologies. Recently, a two-stage topology optimization methodology was proposed for designing multifunctional metamaterials with simultaneous negative thermal expansion and phononic band gap [187], and for designing hybrid metamaterials with simultaneous negative Poisson's ratio and low-frequency band gap [188]. For the latter, unit cells with negative Poisson's ratio are initially determined by optimizing the topological distribution of the matrix material region, while the band gap issue is disregarded. In the second stage, the Poisson's ratio and band gap – induced by adding stiff and soft elements – are considered simultaneously. Here, the band gap objective function was converted into constraints on geometric parameters, reducing the problem to single-objective optimization. Strict constraints and objectives are then applied to refine the Poisson's ratio. This two-stage optimization offers a promising framework that could be extended to other mechanical functionalities.

In contrast, gradient-free optimization bypasses gradient information, relying instead on heuristic methods, random sampling, or direct search strategies to explore the design space. The optimization starts by defining a set of designs represented as discretized domains that respect imposed geometric constraints. Objective functions are evaluated for each design, from which gradient-free methods are employed to update the design variables iteratively. Examples of gradient-free methods used for optimizing mechanical metamaterial designs are genetic algorithms [189], particle swarm optimization [190], simulated annealing [191], and Nelder-Mead method [192]. For simultaneous optimization of mechanical and vibration attenuation properties, single-objective optimization with additional constraints can be applied, whose approach is similar to the second stage of the two-stage optimization method. Another approach is using multi-objective topology optimization, where objective functions related to each desired functionality are derived. As vibration attenuation and mechanical improvement could be two contradictory objective functions, the optimization provides a set of Pareto-front solutions that identifies such tradeoff behavior. The optimization iteratively uses the operations of the gradient-free methods until an optimal Pareto-front solution is obtained, which gives the set of optimized designs with desired functionalities. Such approach has been used to customize in-plane mechanical properties and broadband band gaps via genetic algorithms [189], [193]. Since massive amount of designs is evaluated per objective functions, efforts have been made to mitigate the computational time issue, mainly through parallel computing and model simplifications, such as plain stress and plain strain assumptions, reduced order modeling [194], surrogate models [195], and homogenization techniques [196].

#### 4.1.2 Inverse design process

The step-based and optimization-based forward design processes have in common a creation of a mapping between structural design and desired functionalities. As an alternative for modeling mechanical metamaterials, inverse design approaches have been used to map directly the desired functionalities to the mechanical metamaterial geometry, as indicated in Fig. 6 [197], [198], [199], [200], [201]. This is performed via two neural networks; the first maps geometries into mechanical properties and the second maps mechanical properties into geometries [202]. Data-driven models, such as machine and deep learning, have been applied to link the relation between dispersion curves and structure topology of elastic metamaterials [199], where the forward prediction is performed via convolutional neural network (CNN)s and inverse prediction is performed via conditional generative adversarial network (GAN) (cGANs). When designing hybrid mechanical metamaterials, the mapping dimensionality increases, since a link among static and dynamic properties and structural topology is required. Therefore, more complex inverse design approaches, such as multi-network systems built based on the topology optimization data set [203] could be employed, although with a cost of computational time [204]. In such systems, structural database with distinct material properties is defined by using multi-objective topology optimization algorithm. The generated data set is used to build the multi-network system formed by three networks–GAN, property prediction network (PPN), and structure generation network (SGN)–that will realize the performance evaluation and the structural customization. With respect to HMMMs, the structural database with distinct functionalities could be defined through the multi-objective optimization and further used on the multi-network system.

### 4.2 Further advances in designing HMMMs

The HMMM designs presented previously possess multi-functionalities that cannot be modulated after manufacturing, which could limit their application in case they are exposed to varied external factors, such as different loading conditions or change on vibration bands. Incorporating elements that respond to external stimuli into the metamaterial design allow for active control of static and dynamic properties after fabrication, which could be programmable for specific operational conditions. For instance, metamaterials fabricated with smart materials, such as shape memory polymer (SMP) offer excellent environmental adaptability and high deformation capacity [205], [206], [207]. Topological transformation through heat stimulus in active lattice structures was observed, enabling switching between stretching- and bending-dominated topology [208]. This topological transformation combined with metamaterials with programmable coefficient of thermal expansion [209], [210] could result in unique mechanical performance. Some studies have also demonstrated the tunability of dynamic features through external stimuli, including magnetic field [155], [211], [212], variation of thermal conditions [206], [213], pre-stress induced by shunted piezoelectric patches [214], [215], [216], [217], [218], [219]. For instance, programmable deformation configurations are enabled when combining soft materials with hard-magnetic particles [220]. The different deformation modes resulted from changing of magnetic field and mechanical loading causes the shifting of band gaps [212]. By incorporating smart materials like shape memory polymers or using external factors such as magnetic fields, heat, or pre-stress, these metamaterials can achieve programmable deformation and tunable performance for specific operational conditions. This adaptability enhances their functionality, making them suitable for a wide range of applications where environmental conditions or operational requirements may vary.

Rapid advancements in manufacturing processes enable the integration of such active elements, leading to more intelligent and unified designs. The advent of 3D printing technology significantly broadened mechanical metamaterial design possibilities [221], allowing the exploration of new mechanical properties in designs ranging from micro- to macro-scale [54], [83], [222], [223]. Emerging 4D printing technology [221], [224] integrates multi-physics properties into manufacturing, enabling passive metamaterial features to become active and programmable [225]. These advancements in additive manufacturing will facilitate the rapid implementation of hybrid mechanical metamaterials to address real-world engineering challenges.

### 4.3 Hybrid mechanical metamaterials as solution for engineering problems

The development of hybrid mechanical metamaterials has been motivated by the increase of complexity of engineering problems combined with the advances in computational design methods and manufacturing process. From the unique properties developed by HMMM designs, their potential applications in engineering problems can be drawn and categorized as shown in Table 3.

**Table 3:** Potential application of HMMMs in engineering problems in terms of their unique features.

Feature	Static Property	Dynamic Property	Application
Vibration damping and isolation	Load-bearing capacity	Tunable band gap	1) Infrastructure design: mitigate vibrations caused by seismic activity or machinery [226]
			2) Automotive and aerospace: isolate sensitive components from engine vibrations or mechanical shocks [227]
			3) Machinery and equipment: in industrial settings for reducing vibrational impact on sensitive tools, enhancing precision [228]

Sound insulation and control	Structural integrity	Attenuation or redirection of sound waves	1) Noise barriers: In urban environments and around highways to reduce noise pollution [229]
			2) Underwater acoustics: reduction of noise emission during offshore foundation installation [230], while keeping resistance to high hydrostatic pressure

Wave manipulation and energy harvesting	High durability	Wave propagation control for guiding, trapping or focusing energy	1) Energy harvesting from vibrations: In bridges, railways, buildings, or vehicles where the metamaterial converts mechanical vibrations into electrical energy [231]

Impact and shock absorption	High strength, flexibility or lightweight structure to absorb static loads	High energy absorption capacity under dynamic impacts or shocks	1) Personal protective equipment (PPE): helmets, body armor, and protective clothing that absorb impacts while remaining lightweight and flexible [232]
			2) Crash-resistant structures: in automotive and aerospace industries to absorb energy during collisions, protecting passengers and sensitive components [233]
			3) Sports equipment: helmets, pads, and shoes to enhance performance and safety by absorbing impact forces [158]

Biomedicine	Structural support and stiffness for implants or prosthetics	Tunable mechanical responses and absorb dynamic loads	1) Bone implants: hybrid metamaterials designed to mimic the mechanical properties of natural bone, combining strength with dynamic load absorption [234]
			2) Wearable medical devices: flexible, adaptive materials for devices that monitor and respond to the body's mechanical movements [235]

## 5 Outlook

**Figure 7:**
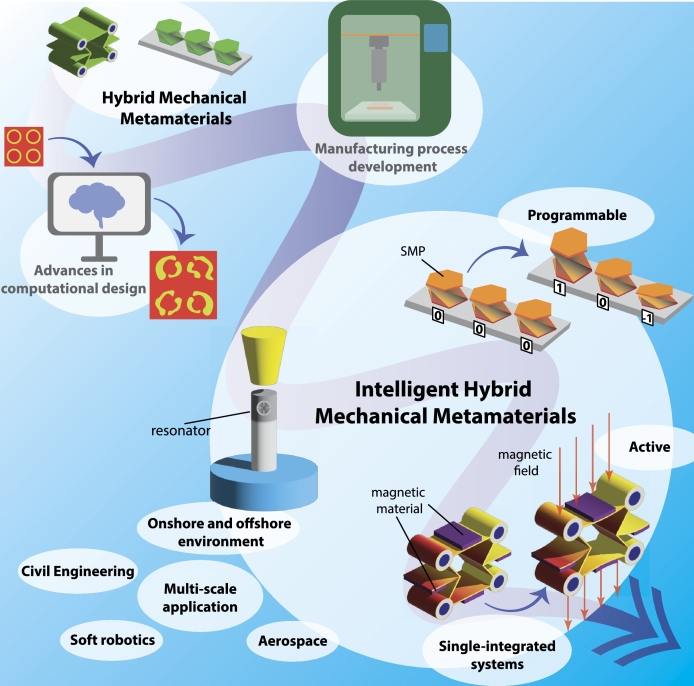
The next generation of hybrid mechanical metamaterials road map.

Beginning with the first local resonant MM presented by Liu et al. (2000) [90] to contemporary hybrid metamaterial designs, there is a discernible trajectory towards increasingly single integrated systems. This trend is poised to persist in the next generation of hybrid metamaterials, albeit with the introduction of new elements aimed at achieving not only multifunctionality but also multi-physics and adaptability capabilities. In essence, the evolution leads towards the development of intelligent HMMMs, as indicated in Fig. 7. Expanding functionalities and incorporating additional physics to the MM models will increase the complexity of theoretical models, numerical simulations, and manufacturing processes. Therefore, future advancements are anticipated to focus on enhancing algorithms and machine learning tools to manage this complexity effectively.

One prospective avenue for intelligent HMMMs involves their integration into real-world applications. Current research has predominantly focused on verifying the functionality of metamaterial designs through numerical simulations, such as a metamaterial-based cushion for noise reduction in offshore installations [230] or laboratory experimental validation [236], [237], [238]. Broader implementation of the next HMMMs in real-world applications hinges on simplifying manufacturing operations. Future investigations could explore various manufacturing techniques, such as 3D printing, laser cutting, metal stamping, or metal forming, to incorporate their mechanical properties, while active and programmable features could be achieved through 4D printing. Furthermore, attention should be directed towards to the life cycle of intelligent HMMMs, aiming to meet engineering demand with minimal impact. Streamlining manufacturing processes will accelerate the integration of intelligent HMMMs into real-world applications, such as engineering problems faced on railways, buildings, and underwater and aerospace devices.

## 6 Conclusions

Metamaterials (MMs) have made significant contributions to vibration control through the manipulation of stiffness-mass ratios in their microstructures – referred to as acoustic/elastic metamaterials (AEMMs) – and to energy absorption, by taking advantage of deformation modes at certain load velocity conditions, mainly quasi-static and low-velocity impacts,– named as mechanical metamaterials (MMMs). However, designing MMs with complex geometries for single tasks may not be conducive to real-world applications, as unforeseen events could necessitate redesigning such MMs with new properties. Additionally, MMs are often designed under controlled environment conditions, which may not accurately reflect the harsh conditions they could encounter in real-world applications. These limitations have prompted researchers to explore novel designs with adaptable multi-functionalities, leading to the emergence of hybrid mechanical metamaterials (HMMMs).

This review sheds light on the development of HMMM designs, which combine the dynamic features of AEMMs with the unconventional static properties of MMMs into a single integrated system. This integration allows for structures that can perform their functions under varying external conditions, although analysis on the effect of high-strain rates on HMMMs is still unexplored. With advancements in computational methods and manufacturing techniques, we anticipate the integration of additional functions from diverse physical principles into future HMMMs. These advancements aim to deliver multidisciplinary, adaptable and fast performance, leading to the emergence of what we term as intelligent HMMMs. Such enhancements in HMMM designs will have the potential to overcome resistance to their implementation in real-world applications.

## CRediT authorship contribution statement

**Ana Carolina Azevedo Vasconcelos:** Writing – original draft, Methodology, Conceptualization. **Dingena Schott:** Writing – review & editing, Supervision, Project administration. **Jovana Jovanova:** Writing – review & editing, Supervision, Project administration, Funding acquisition, Conceptualization.

## Declaration of Competing Interest

The authors declare that they have no known competing financial interests or personal relationships that could have appeared to influence the work reported in this paper.
